# Characterisation of High Alkaline-Tolerant Novel Ulvan Lyase from *Pseudoalteromonas agarivorans*: Potential Applications of Enzyme Derived Oligo-Ulvan as Anti-Diabetic Agent

**DOI:** 10.3390/md22120577

**Published:** 2024-12-23

**Authors:** Navindu Dinara Gajanayaka, Eunyoung Jo, Minthari Sakethanika Bandara, Svini Dileepa Marasinghe, Chinmayee Bawkar, Yeon-Ju Lee, Gun-Hoo Park, Chulhong Oh, Youngdeuk Lee

**Affiliations:** 1 Jeju Bio Research Center, Korea Institute of Ocean Science and Technology (KIOST), Jeju-si 63349, Republic of Korea; navindu@kiost.ac.kr (N.D.G.); jey8574@kiost.ac.kr (E.J.); minthari@kiost.ac.kr (M.S.B.); svini91@kiost.ac.kr (S.D.M.); gunhoopark@kiost.ac.kr (G.-H.P.); 2 Department of Marine Technology & Convergence Engineering, KIOST School, University of Science and Technology, Daejeon 34113, Republic of Korea; chinmayeebawkar@kiost.ac.kr (C.B.); yjlee@kiost.ac.kr (Y.-J.L.); 3 Marine Natural Products Chemistry Laboratory, Korea Institute of Ocean Science and Technology (KIOST), Busan 49111, Republic of Korea

**Keywords:** ulvan lyase, polysaccharide lyase family 25, alpha glucosidase inhibition

## Abstract

Green algae, particularly *Ulva* species, are rich in complex polysaccharides, such as ulvan, which have significant potential for biotechnological applications. However, the biochemical properties of ulvan depolymerised products remain underexplored. The enzymatic depolymerisation of ulvan has garnered attention owing to its cost advantages over alternative methods. Nevertheless, the biochemical characterisation of ulvan lyases, specifically those belonging to the polysaccharide lyase family 25 (PL25), is limited. In this study, we identified and biochemically characterised a novel PL25 ulvan lyase, PaUL25, which functions optimally at pH 10. Additionally, we explored the alpha (α)-glucosidase inhibitory properties of ulvan depolymerised products. PaUL25 exhibited optimum activity at 35 °C in Tris-HCl buffer (pH 10). Moreover, enzyme activity was enhanced by more than 150% in the presence of Mn^2+^ metal ions at and below concentrations of 10 mM. The endolytic action of PaUL25 produced ulvan oligosaccharides with degrees of polymerisation of 2 and 4 as its end products. Partially and completely hydrolysed ulvan oligosaccharides exhibited α-glucosidase inhibitory activity, with half inhibitory concentration IC_50_ values of 3.21 ± 0.13 and 2.51 ± 0.19 mg/mL, respectively. These findings expand our understanding of PL25 and highlight the pharmaceutical potential of ulvan oligosaccharides, particularly as antidiabetic agents.

## 1. Introduction

Marine macroalgae and seaweeds are one of the main components of coastal ecosystems. These organisms are generally attached to rocks or other hard substrates or are free-floating. Marine algae can be classified into three different groups based on the colour of their photosynthetic pigments: red (Rhodophyceae), brown (Phaeophyceae), and green (Chlorophyceae) algae [1]. Marine microalgae/seaweeds play crucial roles in marine ecosystems. Similar to other photosynthetic organisms, seaweeds generate oxygen as a byproduct of photosynthesis [2]. They also provide complex habitats for a diverse array of marine organisms [3] and influence nutrient cycling in marine environments through decomposition and excretion [4]. Over the last few decades, seaweeds have attracted considerable attention owing to their potential applications in various industries, including agriculture, pharmaceuticals, cosmetics, and food [5]. Among the three algae types, green algae have recently gained interest owing to their cell wall composition, growth rate, and versatile biochemical composition [6,7].

Common green algae genera, including *Ulva*, *Enteromorpha*, *Chaetomorpha*, *Codium*, and *Caulerpa* are predominant in the intertidal zones [8]. Among these, *Ulva* spp. are widely distributed across ocean regions, occupying diverse ecological niches [9]. Their ability to rapidly absorb nutrients and proliferate often results in “green tides” [10]. A key feature of *Ulva* spp. is ulvan, a water-soluble sulphated polysaccharide that constitutes approximately 29% of its dry weight [11,12]. Ulvan possesses various biological properties, including anticoagulant [13], antiviral [14], antioxidant [15], antibacterial, antifungal, and anti-inflammatory activities [16]. Structurally, ulvan is composed of linear disaccharides, primarily alpha (α)-linked L-rhamnose-3-sulphate (Rha3S), β-linked glucuronic acid (GlcA), α-linked iduronic acid (IdoA), and β-linked ᴅ-xylose (Xyl) [11]. Major repeating units include ulvanobiuronic acid (Type A: *β-*ᴅ-GlcA [1→4] α-L-Rha3S →1 and Type B: α-L-IdoA [1→4] α-L-Rha3S →1) and minor ulvanobiose units (Rha3S-Xyl) [12]. Additional structural features, including *O*-2 sulfation on xylose, consecutive GlcA residues within the backbone, and branching side chains of GlcA, appear in low abundance [11].

The algal microbiome plays a significant role in algal health, growth, nutrient acquisition, and defence mechanisms, which significantly affect both marine ecosystems and biotechnological applications [17,18,19]. This algal microbiome is not only beneficial in terms of algal vitality and development but also facilitates extensive applications in biotechnology. Marine microbial enzymes, such as lipases, esterases, proteases, and polysaccharide lyases, have numerous industrial applications, including biodiesel production [20], bioremediation [21], food and feed processing [22], and hydrocarbon degradation [23]. Enzymes derived from marine bacteria also facilitate the degradation of various polysaccharides (agar, alginate, cellulose, etc.) [24,25,26], broadening their utility across diverse fields.

According to the Carbohydrate Active Enzyme Database (CAZy; CAZy—Home), significant research has focused on the enzymatic depolymerisation of polysaccharides from brown and red algae, whereas the saccharification process of ulvan from green algae remains comparatively underexplored. The CAZy database categorises ulvan lyases derived from marine bacteria into five different polysaccharide lyase (PL) families: PL24, PL25, PL28, PL37, and PL40. Currently, the PL25 has nine non-homologously characterised ulvan lyases representing different marine bacteria: QFR04505.1 from *Alteromonas* sp. A321 [27], WP_052010178.1 from *Alteromonas* sp. LOR [28], KEZ94292.1 from *Nonlabens ulvanivorans* PLR [29], WP_033186995.1 from *Pseudoalteromonas* sp. PLSV [30] UKQ19338.1 from *Thalassomonas* sp. LD5 [31], SHI30876.1 from *Algibacter luteus* CGMCC [32], VEM00386.1 from *Alteromonas* sp. 76-1 [33], GEA07929.1 from *Alteromonas* sp. KUL42 [34], and WP_120963275.1 from *Alteromonas* sp. TK-46(2) [35]. These enzymes catalyse ulvan depolymerisation through a β-elimination endolytic reaction by cleaving 1–4 glycosidic bonds [27,28,30,31,36]. Given that only a few ulvan lyases from the PL25 family have been biochemically characterised, further investigation of additional ulvan lyases and their potential biotechnological applications is necessary.

Marine polysaccharides and oligosaccharides have garnered significant attention for their diverse potential applications as natural drugs [37], particularly as natural inhibitors of α-glucosidase. This enzyme is in the brush border of the small intestine and plays a crucial role in the final stage of carbohydrate digestion by converting complex carbohydrates into monosaccharides that the body can absorb, thereby increasing blood glucose levels [38]. Inhibiting α-glucosidase controls the release of D-glucose from dietary carbohydrates, effectively moderating postprandial blood sugar spikes [38]. Various marine-derived compounds, such as red algae xylooligosaccharides [39], brown algae-derived compounds [40] and fucoidan [41], and chitosan oligosaccharides [42], have demonstrated α-glucosidase. Marine-derived oligosaccharides are natural alternatives to synthetic drugs like acarbose, with the added benefits of antioxidant and anti-inflammatory properties. Therefore, the discovery and development of novel natural antidiabetic agents hold promise for both pharmaceutical and functional food applications.

The objective of this study was to biochemically characterise a novel ulvan lyase belonging to the PL25, which is distinct from other recognised ulvan lyases. Furthermore, we explored the potential applications of hydrolysed ulvan in the pharmaceutical industry as an antidiabetic agent by confirming its α-glucosidase inhibitory activity. Additionally, we investigated its alkaline condition pH tolerance and functional ulvan lyase activity, enhancing our knowledge of ulvan lyase and its potential applications.

## 2. Results

### 2.1. Screening and Isolation of Ulvan-Degrading Marine Bacteria

The results of serial dilution and streaking on 10% ulvan + seawater plates to identify ulvan-utilising bacteria are shown in Figure S1. Single colonies were screened and isolated on marine agar plates. The 16s rRNA identification revealed that the isolated pale white colony was identified as *Pseudoalteromonas agarivorans*.

### 2.2. Sequence and Phylogenetic Analyses of the Gene paul25

**Figure 1:**
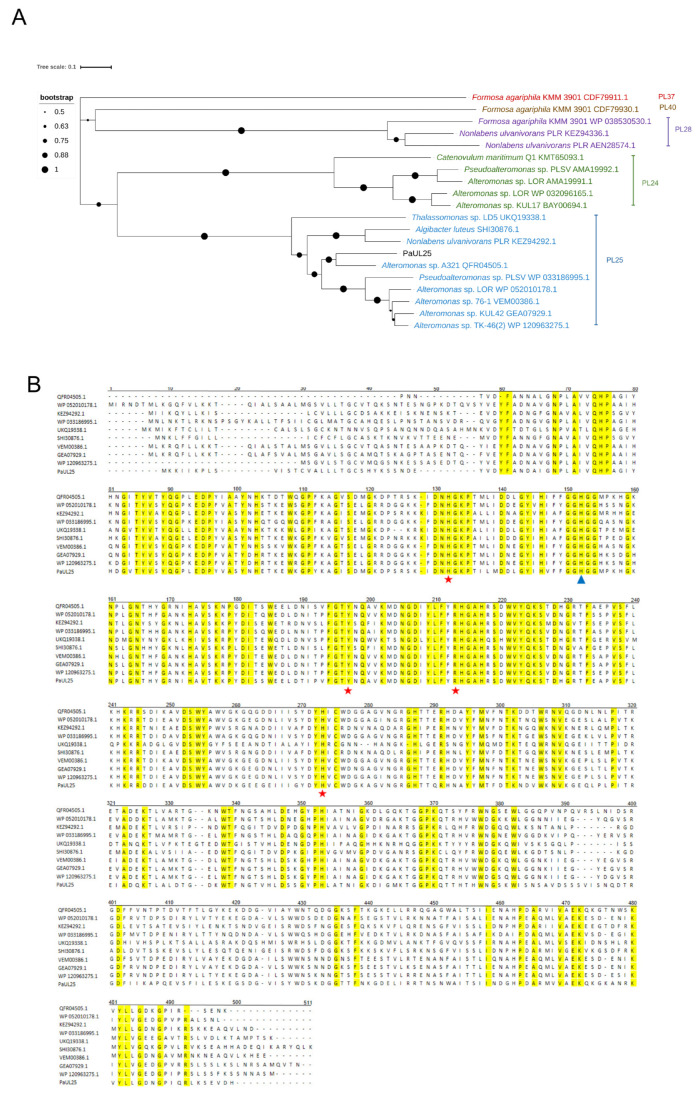
Phylogenetic tree analysis and ulvan lyases gene sequence alignment of PaUL25. (**A**) Phylogenetic tree analysis of PaUL25 with other characterised ulvan lyases that represent different ulvan lyase families. The sequence with the highest similarity, QFR04505.1 (from *Alteromonas* sp. A321), shares 72.6% identity. (**B**) Multiple amino acid sequence alignment between PaUL25 and other ulvan lyases from polysaccharide lyase family 25. Red stars indicate possible catalytic residues, and blue triangles represent possible binding residues of PaUL25. All the data were obtained from the CAZy database. PaUL25, a novel ulvan lyase belonging to the polysaccharide lyase family 25; Carbohydrate Active Enzyme Database.

**Figure 2:**
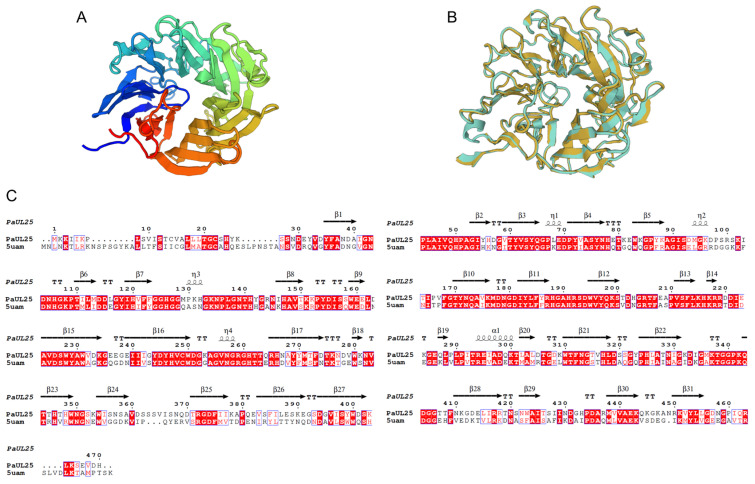
The homologous modelling of PaUL25. (**A**) Predicted structure of PaUL25. (**B**) Structure comparison of PaUL25 (yellow) and PLSV_3936 (cyan). (**C**) Sequence alignment between PaUL25 and PLSV_3936. PaUL25, a novel ulvan lyase belonging to the polysaccharide lyase family 25; PLSV_3936, a previously characterised and structure-determined ulvan lyase.

The PaUL25 sequence was submitted to NCBI under the accession number PQ631038. It contains a 1413 bp open reading frame encoding a protein of 470 amino acids. The predicted theoretical molecular weight and its isoelectric point are 52.61 kDa and 6.54, respectively. The first 21 amino acids constitute the signal peptide. According to the phylogenetic analysis (Figure 1A), PaUL25 harbours a clade of PL25, aligning it with other recorded ulvan lyases in this family. The protein shares its highest sequence identity (72.6%) with the QFR04505.1, an ulvan lyase from *Alteromonas* sp. A321. Multiple gene alignment results revealed five highly conserved amino acids in PaUL25: His_106_, Tyr_171_, Arg_187_, and His_247_, which are crucial for catalytic activity, and His_126_, essential for substrate binding, as observed in PLSV_3936 (Figure 1B). The homology model of PaUL25, generated using SWISS-MODEL, achieved a Global Model Quality Estimation (GMQE) score of 0.83 and a QMEAN z-score of −0.95, indicating a high-confidence model. Sequence alignment with the template structure (5UAM) revealed 59.82% identity, supporting the reliability of the model. The predicted structure of PaUL25 features a seven-bladed β-propeller fold, where each blade consists of four antiparallel β-strands (Figure 2). This structural arrangement was consistent with the typical architecture observed in other ulvan lyases belonging to PL25.

### 2.3. Overexpression and Purification of Recombinant PaUL25

**Figure 3:**
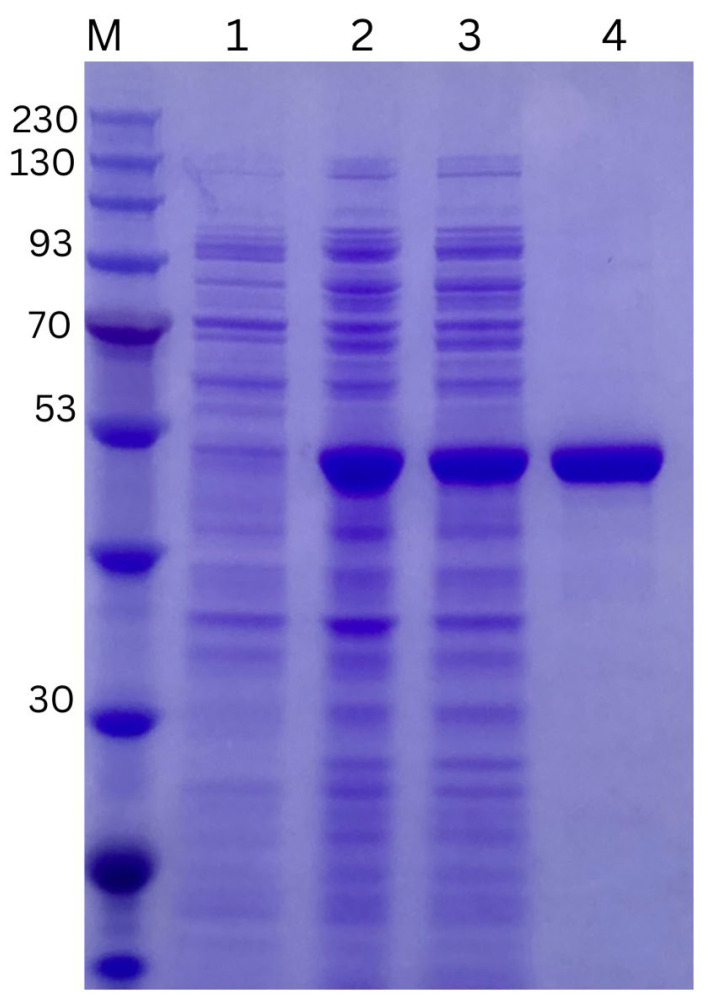
SDS-PAGE (10%, *w*/*v*) analysis of PaUL25. Protein samples were separated on a 10% SDS-PAGE gel and stained with Coomassie brilliant blue. M: Pre-stained molecular mass marker; Lane 1: Total cell lysates before IPTG induction; Lane 2: Total cell lysates after IPTG induction; Lane 3: Total soluble cell lysate after induction; Lane 4: Purified PaUL25. SDS-PAGE, sodium dodecyl sulphate–polyacrylamide gel electrophoresis; PaUL25, a novel ulvan lyase belonging to the polysaccharide lyase family 25; IPTG, isopropyl β-D-1-thiogalactopyranoside.

Recombinant PaUL25 was successfully overexpressed as a soluble protein. SDS-PAGE analysis (Figure 3) illustrates the presence of PaUL25 in different fractions throughout the expression and purification process. Following His tag purification, PaUL25 (lane 4) displayed a prominent single protein band, corresponding to an apparent molecular weight of 50 kDa, which was slightly less than the theoretical molecular weight of 52.61 kDa.

### 2.4. Biochemical Characterisation and Enzyme Kinetics of PaUL25

**Figure 4:**
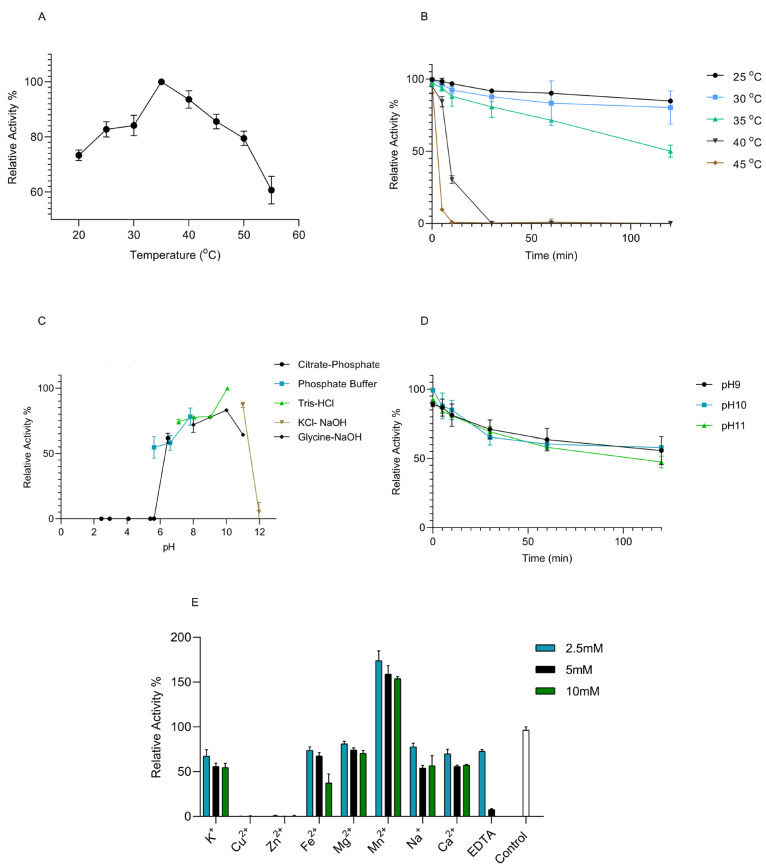
Biochemical characterisation of PaUL25. (**A**) Optimal temperature of PaUL25. Highest activity at 35 °C was set as 100%; (**B**) Thermal stability of PaUL25. Thermal stability was assessed by measuring enzyme activity at 35 °C for 10 min with initial activity set as 100%; (**C**) pH optimisation of PaUL25. Highest activity at pH 10 in Tris-HCl buffer was set as 100%; (**D**): pH stability at 25 °C was assessed by measuring enzyme activity at 35 °C for 10 min with initial activity set as 100%. (**E**): Effect of metal ions and chelate on PaUL25 activity. Activity without metal ion was used as the control and set as 100%. Error bars represent mean ± SD, and all assays were conducted in triplicate (n = 3). PaUl25, a novel ulvan lyase belonging to the polysaccharide lyase family 25; SD, standard deviation.

PaUL25 exhibited optimal activity at 35 °C (Figure 4A), with substantial activity retained at elevated temperatures up to 50 °C. However, temperatures above 50 °C led to a rapid decline in activity. Similarly, temperatures below 25 °C also resulted in reduced activity compared to the optimal activity. Thermal stability tests (Figure 4B) showed that PaUL25 retained consistent or slightly reduced activity at or below 25 °C after 120 min. At 35 °C, activity decreased by approximately 50% after 120 min. Temperatures above 35 °C caused rapid loss of activity within 30 min.

PaUL25 functioned across a broad pH range (5.5–11), with maximum activity observed at pH 10 in the Tris-HCl buffer (Figure 4C). Activity at pH 11 was approximately 80% of the maximum activity. Stability tests (Figure 4D) indicated that PaUL25 retained activity longer at pH 9 and 10 compared to pH 11.

Among the metal ions examined, only Mn^2+^ enhanced PaUL25 activity, increasing it by more than 150% at all tested concentrations (Figure 4E). The other metal ions, including Ca^2+^, Mg^2+^, K^+^, Na^+^, and Fe^2+^, reduced activity to less than 80% of the maximum at all the examined concentrations. Zn^2+^, Cu^2+^, and high EDTA concentrations (10 mM) strongly inhibited activity. Low concentrations of metal ions generally had a more positive effect on PaUL25 activity than high concentrations.

Furthermore, the T_m_ of PaUL25 was determined to be 38.80 ± 0.04 °C, reflecting its thermal stability limits. Kinetic parameters were calculated using the reaction rates across various substrate concentrations. The values for *V_max_*, *K_m_,* and *K_cat_* were 70.21 μmol/min/mg, 22.91 mg/mL, and 58.50/s, respectively. The enzyme efficiency (*K_cat_*/*K_m_*) was calculated as 2.55 mL/mg/s.

### 2.5. End-Product Analysis

**Figure 5:**
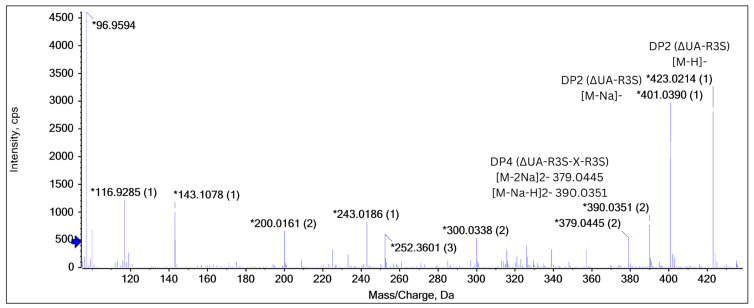
The end-product analysis of PaUL25 using liquid chromatography coupled with mass spectrophotometry (LC-MS). PaUl25, a novel ulvan lyase belonging to the polysaccharide lyase family 25. * referes to the peak identification.

As shown in Figure 5, LC-MS analysis of the ulvan hydrolysate identified several ulvan oligosaccharide products with distinct mass-to-charge (*m*/*z*) ratios. A disaccharide (DP2, ∆UA-R3S) was detected as a doubly charged ion [M − 2H]^2-^ at *m*/*z* 200.0161, a singly charged ion [M − H]^−^ at *m*/*z* 423.0214, and a [M − Na]^−^ at *m*/*z* 401.0389. Additionally, a tetrasaccharide (DP4, ∆UA-R3S-X-R3S) was identified as a doubly charged ion [M − 2Na]^2−^ at *m*/*z* 379.0445 and [M − Na − H]^2−^ at *m*/*z* 390.0351. These results confirm the presence of various ulvan-derived oligosaccharides and successful enzymatic depolymerisation of PaUL25 and show the capability of the enzyme to generate specific oligosaccharide fragments under experimental conditions.

### 2.6. Alpha-Glucosidase Inhibition Assay

**Figure 6:**
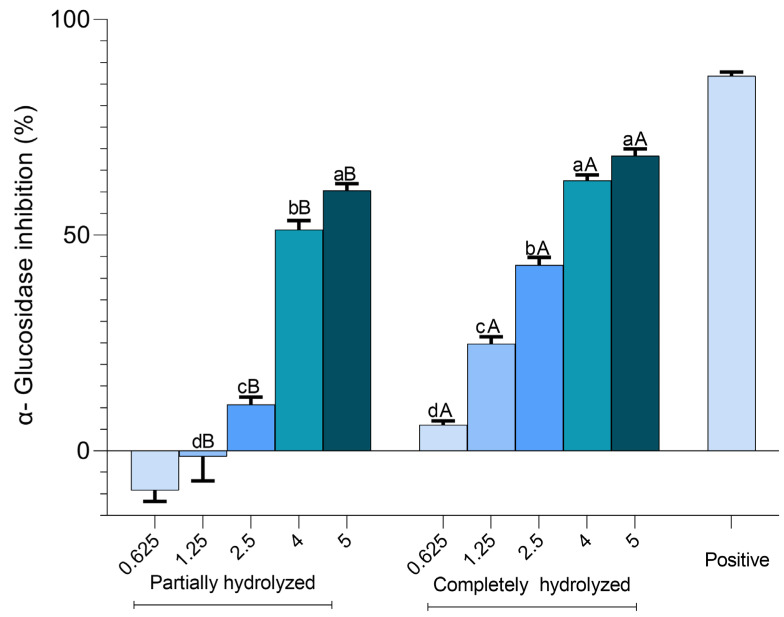
Alpha-glucosidase inhibitory activity of enzyme-derived ulvan oligosaccharides. Data are expressed as the mean ± standard deviation (SD). a–d: means with different letters within the sample group are significantly different (*p* < 0.05). (**A**,**B**): means with different letters at the same concentration across different sample types are significantly different (*p* < 0.05), Acarbose (positive).

Difference concentrations (0.625, 1.25, 2.5, 4, and 5 mg/mL) of partially and completely hydrolysed ulvan were examined for the inhibition of α-glucosidase activity (Figure 6). Completely hydrolysed ulvan at 5 mg/mL showed the highest inhibitory level of 68.36 ± 1.58%. Partially hydrolysed ulvan at the same concentration exhibited a slightly lower inhibition level of 60.31 ± 1.59%. The calculated IC_50_ values for the partially and completely hydrolysed ulvan were 3.21 mg/mL and 2.51 mg/mL, respectively. Two-way ANOVA was performed to evaluate the effect of the hydrolysis state and concentration on the inhibition of α-glucosidase activity. The results revealed a statistically significant effect of hydrolysis type, indicating that the completely hydrolysed samples had significantly higher activity than the partially hydrolysed samples. Furthermore, a significant main effect of concentration was observed, suggesting that changes in the concentration also significantly influenced the inhibition of α-glucosidase activity.

## 3. Discussion

In recent years, advances in molecular biology techniques have led to the identification and characterisation of various ulvan lyases (CAZy—Home). However, the diversity of ulvan lyases in the different polysaccharide lyase families remains underexplored. In this study, we introduce a novel ulvan lyase, PaUL25, which belongs to PL25, thereby expanding existing knowledge of PL25. Notably, PaUL25 is the first documented ulvan lyase to exhibit optimal activity at pH 10. Although no prior studies have highlighted highly alkali-tolerant ulvan lyases for industrial applications, the discovery of such enzymes presents exciting prospects for industries operating in highly alkaline environments.

To date, nine ulvan lyases belonging to PL25 have been identified. However, only five have been comprehensively biochemically analysed [27,31,33,34,35]. Therefore, cross-family comparisons remain limited. Molecular weight comparisons across families suggests that PaUL25, with a molecular weight of 50 kDa, is the smallest among the recorded PL25 ulvan lyases, which include ULA-2 (54 kDa), ALT3695 (53 kDa), TsUly25B (54.54 kDa), LOR_29 (52 kDa), NLR_492 (55 kDa), and ULA-3 (54.39 kDa). Additionally, PaUL25 exhibited the lowest optimum activity temperature at 35 °C, compared to the typical optimum temperature range of 45–60 °C for other PL25 ulvan lyases.

Furthermore, previous studies have demonstrated the effects of Mn^2+^ on PL25 ulvan lyases. For instance, Mn^2+^ reduced activity to 69.89% [34] and 83.90% [35] at a concentration of 1 mM, while enhancing ULA-3 activity by 118.87% [33] at the same concentration. However, Mn^2+^ enhanced the activities of PaUL25 by 173.85%, 158.98%, and 153.99% at concentrations of 2.5, 5, and 10 mM, respectively. Therefore, Mn^2+^ can influence the activity of different PL25 ulvan lyases by either enhancing or reducing enzyme function. Additionally, previous studies have shown that Cu^2+^ and Zn^2+^ decrease or inhibit the activity of PL25 ulvan lyases [27,33,34,35]. Similar results were observed in the present study, where Cu^2+^ and Zn^2+^ inhibited PaUL25 activity. EDTA, a chelating agent, can reduce the activity of ulvan lyases at low concentrations (1–5 mM) [27,33,34,35] and inhibit their activity at high concentrations (10 mM) [27]. Similar to these findings, EDTA decreased PaUL25 activity at 2.5 and 5 mM and inhibited it at 10 mM.

End-product analysis of PL25 ulvan lyases revealed the production of oligosaccharides with varying degrees of polymerisation (DP), with the lowest observed DP being 2. Mass spectrophotometry analysis revealed that the products predominantly consisted of a mixture of disaccharides (Rha3S-GlcA), trisaccharides (Rha3S-Xyl-Rha, Rha3S-lduA-Rha), and tetrasaccharides (Rha3S-lduA-Rha3S-Xyl, Rha3S-lduA-Rha3S-Xyl2s) [27,31,33,34,35]. Consistent with these findings, PaUL25 produces oligosaccharides with DP values ranging from 2 to 4 as its main end products.

Alpha-glucosidase inhibitors are a group of drugs commonly used to treat type 2 diabetes, either as a monotherapy or in combination with other antidiabetic agents. They have also shown benefits in patients with impaired glucose tolerance [43]. In recent years, marine algae and their derivatives have emerged as potential natural sources of antidiabetic compounds owing to their diverse biochemical properties [44]. This study is the first to report the α-glucosidase inhibitory potential of partially and completely hydrolysed ulvan. While the examined ulvan polysaccharide YU11689 from Biosynth Ltd. exhibited no α-glucosidase inhibition during the assay, partially and completely hydrolysed ulvan demonstrated inhibitory effects (Figure S2). These findings underscore the potential of low-molecular-weight ulvan, produced via ulvan lyases, to enhance the antidiabetic properties of ulvan. Further in vivo and animal model experiments are warranted to elucidate the mechanisms underlying α-glucosidase inhibition by ulvan derivatives.

In conclusion, this study presents PaUL25, a novel ulvan lyase from *P. agarivorans* PUA1002. This enzyme functions optimally at 35 °C, pH 10 in Tris-HCl buffer and, retaining more than 90% activity after 2 h at temperatures below 25 °C. Additionally, ulvan oligosaccharides produced by PaUL25 demonstrate α-glucosidase inhibitory activity, suggesting potential development into a type 2 antidiabetic drug. These findings enhance our understanding of PL25 ulvan lyases and open new avenues for industrial applications of ulvan-derived depolymerised products, particularly in the pharmaceutical context.

## 4. Materials and Methods

### 4.1. Media Preparation, Screening and Isolation of Ulvan-Degrading Marine Bacteria

Crude ulvan was extracted using the hot water extraction method [45,46] with minor modifications. A 5% powdered Ulva solution was placed in a thermostatic bath at approximately 85 °C for 3–4 h to dissolve all water-soluble components. The insoluble *Ulva* fraction was separated through centrifugation (7000× *g* for 20 min at 24 °C). The supernatant was precipitated overnight with three volumes of 94% ethanol at cold temperature (10 °C). The resulting pale white precipitate was filtered through a nylon cloth and freeze-dried to a constant weight. The crude ulvan was finely powdered for media preparation. A 10% (*w*/*v*) ulvan solution was combined with seawater and used to prepare ulvan media plates.

Partially decomposed Ulva species-associated water samples (50 mL) were collected from Seongsan Beach, Jeju Island, Republic of Korea (September 2022). These samples were thoroughly crushed and vortexed to homogenise the distribution of microorganisms. Then, the samples were diluted 10 and 100 times with autoclaved seawater. A 70 μL aliquot of each diluted sample was evenly spread on 10% crude ulvan media plates using autoclaved glass beads and incubated at 25 °C for 1–3 days until noticeable changes were observed on the plates. The samples were subcultured on marine agar plates until pure cultures were obtained. The 16S rRNA gene sequences of the observed colonies were amplified for species identification.

### 4.2. Sequence and Phylogenetic Analyses of the Gene paul25

The draft whole-genome sequence (coverage 542.09×) of *Pseudoalteromonas agarivorans* PUA1002 was obtained from CJ Bioscience, Inc. (Seoul, Republic of Korea). The putative ulvan lyase gene, which belongs to the PL25, was identified using the Basic Local Alignment Search Tool (BLAST) and CAZy database. The identified gene, *paul25*, was submitted to the NCBI GenBank. Conserved domain structure analysis was conducted using SMART version 9.0 [47] and the InterPro tool version 97.0 [48]. The predicted signal peptide was determined using the web tool SignalP version 6.0 [49]. The theoretical value of molecular weight (Mw) and isoelectric point (pI) were computed using the web tool Expasy Server [50]. The multiple sequence alignment of available PL25 ulvan lyases and a phylogenetic tree were constructed using MEGA11 (Molecular Evolutionary Genetics Analysis version 11) software [51]. The Interactive Tree of Life (iTOL) version 6 [52] web-based tool was used to enhance the visualisation of the phylogenetic tree. Protein structure modelling for PaUL25 was conducted using the SWISS-MODEL web tool, with PDB ID 5UAM serving as the template for protein structure prediction.

### 4.3. Overexpression and Purification of Recombinant PaUL25

The *paul25* gene was cloned without the signal peptide. The genomic DNA from *P. agarivorans* PUA1002 was isolated using the AccuPrep^®^ Genomic DNA Extraction Kit (Bioneer, Daejeon, Republic of Korea), following the manufacturer’s protocol. The extracted DNA was used as a template for amplification. The forward primer 5′-ATCGAAGGTCGTCATATGTATAAATCGAGTAATGATGAGTATGTAGATTACTTTGCC-3′ and reverse primer 5′-TTGTTAGCAGCCGGATCCTTAATGATCTACTTCAGACTTTAAACGCTG-3′ were used for the amplification of the *paul25*. The amplified gene with His tag was ligated into the pET-16b vector using the Ez-Fusion ^TM^ Cloning Kit (Enzynomics, Daejeon, Republic of Korea). Restriction enzymes Nde I and BamH I were used to linearise the vector. The completed vector was transformed into *Escherichia coli* (DH5α) and cultured on Luria–Bertani (LB) ampicillin (LB/amp) media plates overnight at 37 °C. A single colony was used to inoculate 4 mL of LB/amp, which was cultured overnight at 37 °C with shaking at 180 rpm. Positive transformants were verified by sequencing (Macrogen, Seoul, Republic of Korea) the extracted plasmids using the AccuPrep*^®^* Plasmid Extraction Kit (Bioneer). After verification, the extracted plasmids were transformed into *E. coli* BL21(DE3) cells for expression studies. Gene expression was induced at an optical density (OD_600_) of 0.6–0.8 using a final concentration of 0.1 mM isopropyl-β-D-thiogalactopyranoside (IPTG). The culture was incubated at 16 °C, 180 rpm for 16–18 h in LB/amp medium. Subsequently, the cells were harvested by centrifugation at 8000× *g* for 10 min at 4 °C and resuspended in column buffer for His tag purification. The cells were disrupted using sonication, and the soluble protein fraction was separated by centrifugation at 13,000× *g* for 30 min at 4 °C. A Nova-gen His Tag Purification Kit (Nova-gen, Madison, WI, USA) was used to purify PaUL25, according to the manufacturer’s instructions. The protein purity was analysed using sodium dodecyl sulphate polyacrylamide gel electrophoresis (10%, *w/v* SDS-PAGE). A bicinchoninic acid (BCA) protein assay kit (Thermo Fisher Scientific Inc., Waltham, MA, USA) was used to determine the concentration of the purified PaUL25.

### 4.4. Biochemical Characterisation and Enzyme Kinetics of PaUL25

The temperature optimisation for PaUL25 activity was examined within the range of 20–55 °C. The thermal stability of PaUL25 was assessed by pre-incubating the purified enzyme at various temperatures (25 °C, 30 °C, 35 °C, 40 °C, and 45 °C) for different durations (5 min, 10 min, 30 min, 60 min, and 120 min). The optimum pH of PaUL25 was determined by exposing the enzyme to different pH buffers and ranges: 50 mM citrate–phosphate buffer (pH 3–7), 50 mM phosphate buffer (pH 6–8), 50 mM Tris-HCl buffer (pH 7–10), 50 mM glycine–NaOH buffer (pH 8–11), and 50 mM KCl-NaOH buffer (pH 11–12). Additionally, the pH stability was examined by incubating the enzyme at 25 °C within a pH range of 9–11 for 120 min. Finally, the influence of metal ions and chelates on PaUL25 activity was determined using different metal ions (Ca^2+^, Mg^2+^, K^+^, Mn^2+^, Zn^2+^, Cu^2+^, Na^+^, and Fe^2+^) and EDTA at different concentrations (2.5, 5, and 10 mM). For all characterisation assays, 1% (*w*/*v)* crude ulvan was used as the substrate, extracted via the hot water extraction method [46]. The melting temperature (T_m_) of the purified PaUL25 was determined using the UNcle-protein stability screening platform (Unchained Laboratories, Pleasanton, CA 94588).

Commercial ulvan powder (YU11689) from Biosynth Ltd. (Staad, Switzerland) was used for enzyme kinetic experiments. Substrate concentrations ranging from 0 to 40 mg/mL were used to determine the kinetic parameters under optimum temperature and pH conditions over a 10 min reaction time. L-rhamnose served as the standard for the assay. Ulvan lyase activity was defined as the amount of enzyme required to produce 1 μmol of reducing sugars per minute. Kinetic parameters, including *V_max_*, *K_m_*, *K_cat_*, and catalytic efficiency (*K_cat_*/*K_m_*), were determined using the allosteric sigmoidal model in GraphPad Prism version 10.3.0 (GraphPad Software, Inc., San Diego, CA, USA). Enzyme activity was quantified by the dinitrosalicylic acid (DNS) method, with all reactions terminated prior to measurement [53].

### 4.5. End-Product Analysis

To analyse the end products produced by PaUL25, the enzyme reaction mixture was prepared by incubating purified PaUL25 with a 1% (*w*/*v*) solution of commercial ulvan for 24 h. Following incubation, the reaction mixture was filtered through a 0.45 μm filter and diluted to a concentration of 10 ppm (0.001%) using MS-grade water and subjected to liquid chromatography coupled with mass spectrophotometry (LC-MS). A 10 μL aliquot of the diluted sample was injected into a Phenomenex Kinetex C18 column (2.1 × 100 mm, 2.6 µm, 100 Å) for chromatographic separation. The separation was performed under isocratic conditions using 90% aqueous methanol as the mobile phase. Mass spectrometric analysis of the reaction products was conducted using an X500R QTOF system (SCIEX, Framingham, MA, USA) in the negative ion mode. The obtained data were processed to determine the molecular mass and fragmentation patterns of the ulvan hydrolysate products, providing insight into the enzymatic cleavage patterns and the specific end products generated by PaUL25.

### 4.6. Alpha-Glucosidase Inhibition Assay

The α-glucosidase inhibitory activity of partially and completely hydrolysed ulvan samples was examined using the α-glucosidase inhibitor screening kit (ab284520; Abcam, Cambridge, UK) according to the manufacturer’s protocol. The reaction mixture was prepared with 10 μL of different concentrations of partially hydrolysed ulvan samples and completely hydrolysed ulvan samples, 10 μL of α-glucosidase enzyme solution, and 60 μL of assay buffer in a 96-well plate. Each sample was thoroughly mixed and incubated for 15 min at room temperature in the absence of light. Subsequently, 20 μL of substrate mixture was added to each well and mixed thoroughly. The absorbance was measured at 410 nm in kinetic mode at 25 °C for 60 min using a microplate reader (Multiskan™ GO, Thermo Fisher). The IC_50_ values were calculated based on nonlinear regression analysis, and two-way ANOVA was performed to evaluate the effect of the hydrolysis state and concentration on the inhibition of α-glucosidase activity using GraphPad Prism version 10.3.0 (GraphPad Software, Inc.).

## Data Availability

The original contributions presented in the study are included in the article/Supplementary Materials. Further inquiries can be directed to the corresponding author.

## Supplementary Materials

The following supporting information can be downloaded at: https://www.mdpi.com/article/10.3390/md22120577/s1, Figure S1: Ulvan-utilising ability of *Pseudoalteromonas agarivorans*. Figure S2: Alpha-glucosidase inhibitory activity of enzyme derived ulvan oligosaccharides.
