# Supplementary material for: Characterisation of High Alkaline-Tolerant Novel Ulvan Lyase from Pseudoalteromonas agarivorans: Potential Applications of Enzyme Derived Oligo-Ulvan as Anti-Diabetic Agent
Source: Mar Drugs. 2024 Dec 23;22(12):577. doi: 10.3390/md22120577 (PMC11676845; doi:10.3390/md22120577)
Supplement: Supplementary file 1 [file marinedrugs-22-00577-s001.zip › marinedrugs-3357917-supplementary.pdf]

Supplementary Materials

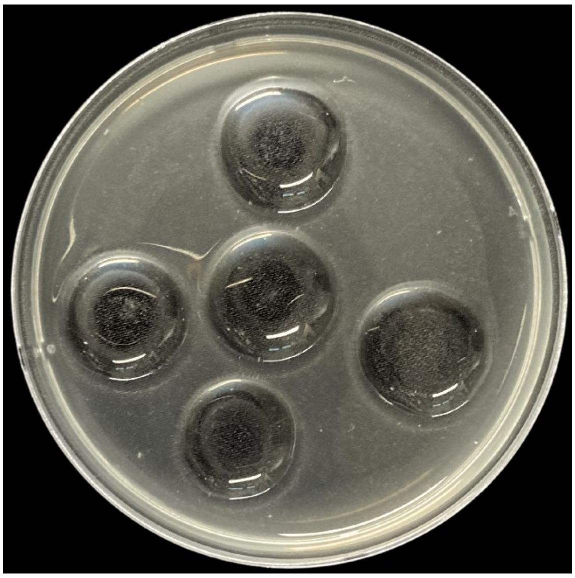

Figure S1. Ulvan-utilizing ability of *Pseudoalteromonas agarivorans*. Plate containing 10% ulvan + filtered seawater and was incubated at room temperature for 3-4 days.

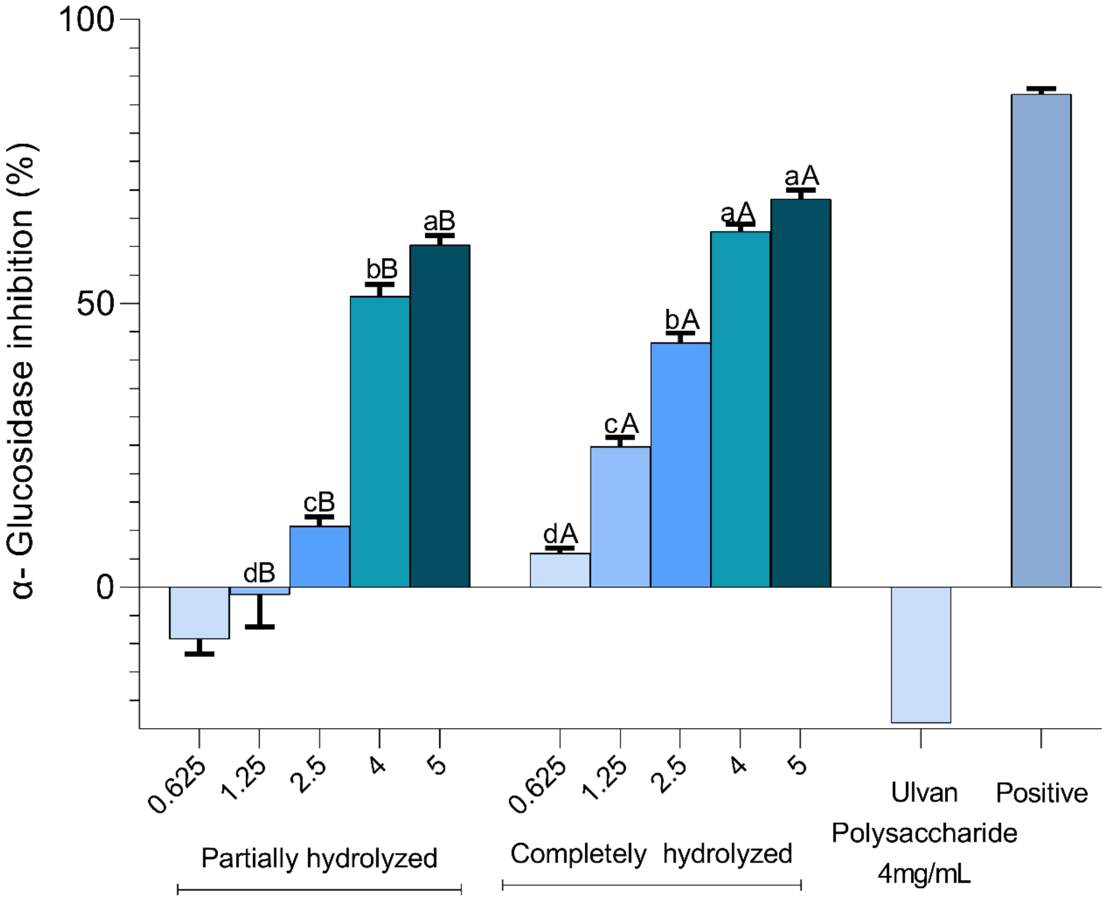

Figure S2. Alpha-glucosidase inhibitory activity of enzyme-derived ulvan oligosaccharides. Data are expressed as the mean ± SD. Different lowercase letters (a–d) within the same sample indicate significant differences ($p < 0.05$). Different uppercase letters (A–B) at the same concentration among different sample types indicate significant differences ($p < 0.05$).
